# Axillary Metaplastic Breast Carcinoma with Ipsilateral Pectoral Invasive Ductal Carcinoma: An Unusual Presentation

**DOI:** 10.1155/2014/938509

**Published:** 2014-09-16

**Authors:** Lei Zhang, Sabahattin Comertpay, David Shimizu, Richard M. DeMay, Michele Carbone, Stacey A. Honda, Jodi M. Matsuura Eaves

**Affiliations:** ^1^Department of Pathology, University of Hawaii, 651 Ilalo Street, No. 411E, Honolulu, HI 96813, USA; ^2^Mercy Hospitals at Bakersfield, 2215 Truxtun Avenue, Bakersfield, CA 93301, USA; ^3^Cancer Center of Hawaii, 2226 Liliha Street, Honolulu, HI 96817, USA; ^4^Department of Pathology, University of Chicago, 5841 South Maryland Avenue MARP 212, MC 2050, Chicago, IL 60637, USA

## Abstract

We report a case of axillary metaplastic breast carcinoma (MBC) with triple negative (ER−/PR−/Her2−) phenotype, concurrent with multifocal invasive ductal carcinoma (IDC) of ipsilateral pectoral breast (ER+/PR+/Her2−) in a 60-year-old woman. The two tumors demonstrate different morphology, immunophenotype, and opposite response to neoadjuvant chemotherapy of paclitaxol, adriamycin, and cyclophosphamide. Methylation analysis of human androgen receptor (HUMARA) on X-chromosome identified monoclonal pattern of X-chromosome inactivation in MBC and mosaic pattern in the IDC. Stem cell origin of MBC is suggested in this case. Clinicopathological features, imaging findings, biological markers, chemoradiation management, and prognosis of MBC are reviewed in comparison to invasive ductal carcinoma. Our case and literature review suggest that traditional chemotherapy applicable to IDC is less effective towards MBC. However, new chemotherapy protocols targeting stem cell and multimodality management of MBC are promising. Recognition of unusual presentation of MBC will help tailor therapy towards tumor with worse prognosis.

## 1. Introduction

Metaplastic breast carcinoma (MBC) constitutes 0.2–1% of all breast cancer diagnoses [1, 2]. Axillary MBC has not been described. We present a rare case of ipsilateral synchronous MBC in the axillar with concurrent multifocal invasive ductal carcinoma (IDC) of pectoral breast and discuss clinicopathological and management differences of these two tumors.

## 2. Case Presentation

### 2.1. Clinical Presentation

A 60-year-old female with no family history of malignancy presented with a left breast mass and left axillary enlargement. Computed tomography (CT) and magnetic resonance imaging (MRI) of the chest showed two masses in the left breast: one at 9 o'clock, 1 cm from nipple and 3.4 cm in size, and the other at 2 o'clock, 7 cm from nipple and 1.6 cm in size. A left axilla mass measuring 5.4 cm with smooth border suspicious for lymph node was also identified. CT of the chest and abdomen showed no other metastases. The 9 o'clock lesion was biopsied and the patient was treated with 12 cycles of Taxol and 4 cycles of adriamycin and cyclophosphamide. The medial and lateral lesions of left breast both shrank, but the axillar mass enlarged.

Modified radical mastectomy and axillary dissection were performed at the earliest possible time. Shortly after the surgery, lung and brain metastases were identified and the patient elected to hospice discharge and subsided after 10 months.

### 2.2. Pathological Findings

**Figure 1:**
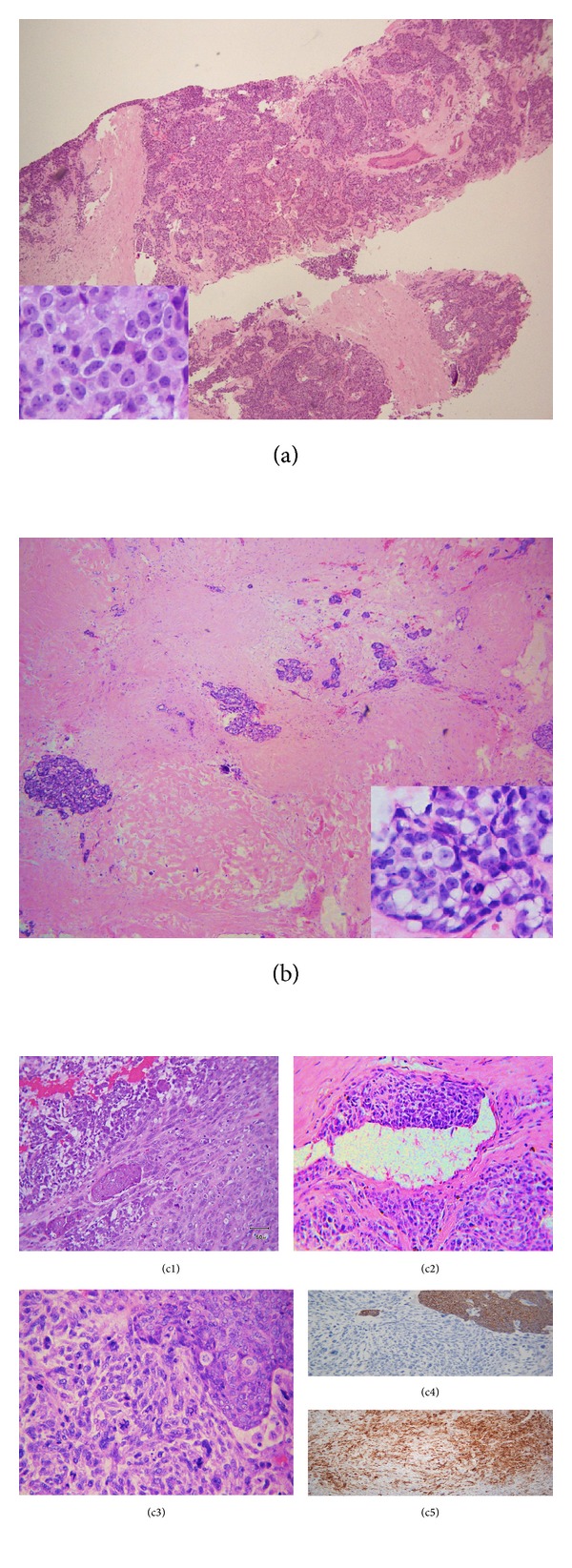
Invasive ductal carcinoma in the left breast before (a) and after (b) neoadjuvant therapy and axillary metaplastic breast carcinoma (c). (a) Cellular tumor clusters with poor tubular formation (×20), moderate pleomorphism and occasional mitosis (×100 inset). (b) Tumor islets separated by fibrosis (×20), composed of cells with cytoplasmic empty vacuoles and occasional pyknosis consistent with therapy effect (×100 inset). (c1) Intertwining spindle cells, adenocarcinoma, and squamous cell carcinoma (×40). (c2) Vascular invasion (×20). (c3–c5) Spindle and poorly differentiated squamous components with different stains. (c3) HE (×40); (c4) AE1/AE3 (×40); (c5) smooth muscle myosin heavy chain (×40).

Biopsy of the 9 o'clock breast lesion showed invasive ductal carcinoma (Figure 1(a)), positive for estrogen receptor (ER, 100%) and progesterone receptor (PR, 24%) and negative for HER2 overexpression. Histologic examination of radical mastectomy and axillary dissection specimen confirmed partial chemotherapy response of invasive ductal carcinoma with residual tumor foci of 1.5 cm at 9 o'clock and 0.9 cm at 2 o'clock (Figure 1(b)).

The axillary mass measured 13 × 12.5 × 7 cm. The axillary tumor did not involve skin and was not connected to the pectoral breast lesions. The axillary tumor showed mixed adenosquamous and spindle cell components with high mitotic rate (Figures 1(c1) and 1(c3)). The tumor invaded vessels (Figure 1(c2)), but no discrete lymph node was identified. Both spindle and poorly differentiated squamous components were immunoreactive for smooth muscle myosin heavy chain (Figure 1(c4)), while the pancytokeratin AE1/AE3 expression was limited to epithelial component (Figure 1(c5)), supporting the diagnosis of metaplastic carcinoma.

The pathological diagnoses were (1) invasive ductal carcinoma of pectoral breast, grade 2, ypT1c, with partial chemotherapy response, ER+/PR+/Her2−, and (2) axillary breast metaplastic carcinoma with vascular invasion, grade 3, ypT3, ER−/PR−/Her2−.

### 2.3. Genetic Findings

**Figure 2:**
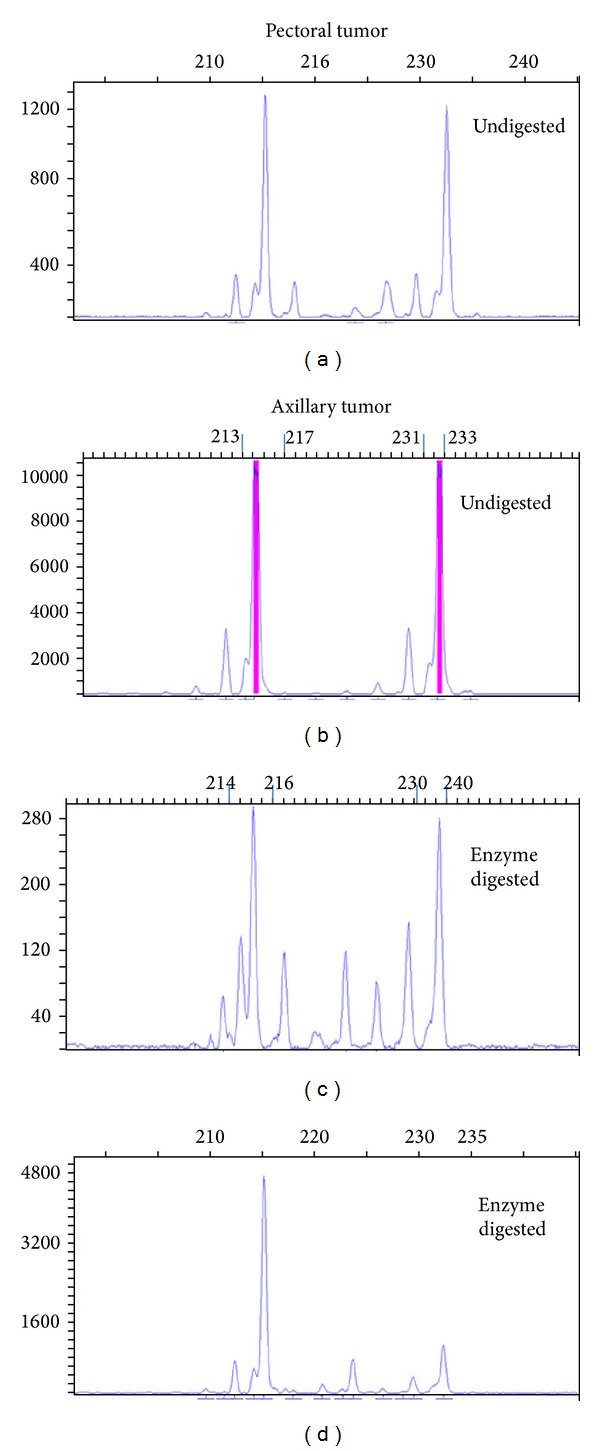
Fluorescent peak trace chromatogram of pectoral breast carcinoma and axillary metaplastic breast carcinoma. Pectoral invasive ductal carcinoma. (a, c) Polyclonal profile in the capillary electrophoresis characterized by the persistence of two distinct allele peaks after HpaII digestion. The additional smaller peaks are caused by slippage of the Taq polymerase. Axillary metaplastic breast carcinoma. (b, d) Complete disappearance of one allele peak after enzymatic digestion suggestive of monoclonal pattern.

To analyze genomic similarity of those tumors, we performed X-chromosome linked methylation analysis of human androgen receptor (HUMARA), using dissected formalin fixed paraffin embedded tissue. The principals are based on (1) the presence of 9 to 36 CAG short tandem repeats (*n* = 9 to 36) located in the first exon of human androgen receptor on X-chromosome; length variation in CAG repeats enables separation of maternal and paternal chromosome in 90% of females; (2) the presence of cleavage site for methylation-sensitive restriction enzyme HpaII in close proximity to the CAG repeat. This site is randomly inactivated (methylated) in early embryogenic development. As such, the methylation ratio of maternal and paternal chromosome in normal somatic tissue would be 1 : 1. Any significant deviation (>3 : 1 or <1 : 3) indicates clonal expansion. Briefly, the extracted DNA was subjected to HpaII digestion (methylated site will not be cut) and mock digestion, followed by PCR amplification. The PCR products were then run through capillary electrophoresis (Applied Biosystem Bioanalyzer) and data output as the fluorescent peak trace chromatogram. The biopsied and surgically resected foci of pectoral tumor at 9:00 both showed a polyclonal mosaic pattern (Figures 2(a) and 2(c)), while the axillary tumor was monoclonal (Figures 2(b) and 2(d)). This suggests that the two tumors bear different genetic signatures.

## 3. Discussion

The early diagnosis of axillar MBC is difficult in this case because of the unusual location and deceptive lymph node-like imaging finding in the presence of pectoral IDC. It is particularly difficult to differentiate ectopic breast cancer from lymph node metastasis by imaging studies alone. The distinction relies primarily on morphology, supported by special studies. Morphologic and genetic studies were concordant in making a diagnosis of synchronous tumors in this patient.

Surgery is a common approach performed in 96.5% MBC patients with 55.5% patients receiving mastectomy and 41.0% having lumpectomy [3]. Meta-analysis revealed no difference in overall survival (OS) between these two surgical types [3]. Of note, 38.6%–49.1% MBC patients have postsurgical radiotherapy (RT). Although the OS in patients receiving RT is 60.3% at 10 years compared to 48.3% in patients not receiving RT, there is a significantly better survival rate for patients receiving postlumpectomy RT (16.2%) compared with postmastectomy RT (2.4%) (Table 1). The reason could be that the patients selected for lumpectomy have a smaller mass and RT works better for minimizing local recurrences in smaller MBC tumor. This has made postlumpectomy RT a standard component of MBC management.

Comparing to IDC, MBC is larger in size with lower rate of lymph node metastasis and more frequent hematogenous spread (Table 1). The larger size at presentation has made mastectomy an optimal choice for 55.5% of MBC patients [3]. The moderate 2.4% benefit of postmastectomy RT and frequent hematogenous metastasis make patients a candidate for chemotherapy. Although MBC patients receive chemotherapy more frequently, the response to conventional chemotherapy is poor compared to IDC. There is also no response difference between neoadjuvant and adjuvant therapy (Table 1). This is in contrast with triple negative breast carcinoma (TNBC), in which neoadjuvant chemotherapy is associated with improved survival compared with adjuvant chemotherapy [4]. Meta-analysis showed that the improved survival is present only after pathological complete response (pCR) [4]. The absence of response difference in the timing of chemotherapy in MBC is most likely due to the lack of pCR. This indicates that the decision of neoadjuvant versus adjuvant chemotherapy may not be as critical as early surgery if no pCR can be achieved. New chemotherapy protocol being able to achieve pCR is needed and understanding of the pathogenesis of MBC may help in novel regimen development.

MBC is distinct from IDC morphologically by the presence of additional metaplastic squamous or mesenchymal components. Clonality analysis using HUMARA revealed that various components in MBC are clonally related in 100% (4/4) of cases [5]. Unexpectedly, morphologically homogeneous IDC showed monoclonal pattern only in 67% (8/12) of cases [6]. The different clonal pattern is once again observed in concurrent MBC and IDC in our case. Pluripotential stem cell origin is implied in MBC. Concordantly, stem cell related Wnt/*β*-catenin [7] and PIK3CA/PTEN → mTOR/hypoxia-inducible factor (HIF) → vascular endogenous growth factor (VEGF) signal pathways [8] which drive epithelial to mesenchymal transition and angiogenesis are upregulated in MBC (Table 1). The overexpression of EGFR in MBC may be related to squamous differentiation (Table 1) [9].

A new protocol targeting cancer stem cells composed of antiangiogenic antibody Bevacizumab and the mTOR/hypoxia-inducible factor inhibitor Temsirolimus combined with antiproliferation liposomal doxorubicin has shown complete/partial pathological response as an adjuvant chemotherapy in phase I clinical trials in 2/5, 40% [10], and 5/12, 42% [11], of patients with a diagnosis of MBC.

Most MBC is ER/PR/HER2 triple negative and TNBC is enriched in stem cell characteristics. Therapies effective in TNBC may shed light on the therapy of MBC. Meta-analysis on the pCR of TNBC towards neoadjuvant chemotherapy reveals that the platinum-containing group had a higher pCR of 44.2% than either the anthracycline-based (26.8%) or taxane-containing (30.5%) groups (Table 1) [4]. Accordingly, adding cisplatin to taxane/anthracycline/cyclophosphamide adjuvant chemotherapy regimen has decreased the relapse rate of MBC from 56% (10/18) to 44% (4/9) in one study (Table 1) [1]. Timely surgery followed by carboplatin and albumin-bound paclitaxel has achieved complete remission in one patient with tumor emboli and recurrent chondroid-metaplastic breast cancer [12].

Epithelial growth factor receptor (EGFR) is overexpressed in MBC compared to IDC (Table 1). This could suggest a potential therapeutic benefit of protein kinase inhibitor gefitinib [2, 9].

In our case, pectoral IDC showed partial pathological response and clear margins after surgery. The major concern is the axillary MBC which is resistant to the conventional chemotherapy, demonstrating rapid growth, vascular invasion, and positive surgical margin. In lieu of the reported very moderate effect of postmastectomy RT [3] and suggested stem cell origin of MBC based on morphology and HUMARA study, this patient could be a candidate for contemporary adjuvant chemotherapy with stem cell targeting, while RT might be also considered for local control.

MBC is shown to have poorer prognosis than TNBC and IDC (Table 1) [1, 13]. However, a 5-year survival of 80% is reported in a recent case series [14]. Comparing to other MBCs shown in Table 1, those cases are characterized by (1) young age with mean of 46 years (range 26–66); (2) smaller tumor at diagnosis with median size of 3.5 cm (range 1.5–12 cm) and 24% of tumor < 2 cm and 47% of tumor between 2.0 and 5.0 cm; (3) all tumors being larger than 5 cm (mean 9.0 cm, range 7.0–12.0 cm), happening to occur in young women (mean 35 years, range 30–40 years), and demonstrating a 5-year disease free survival of 75% (3/4). In this series, lumpectomy and mastectomy were performed in 35% and 65% of patients, respectively. All patients have postoperative radiotherapy and adjuvant chemotherapy. The chemotherapy is conventional and composed of doxorubicin/cyclophosphamide (59%) and taxane (35%), similar in our patient. Lymph node metastasis rate is 35% in this series. However, lymph node status has been suggested not to affect prognosis in MBC [13]. Histologically, 94% (16/17) of tumors are grade 3 in this series. There is no low grade fibromatosis-like or low grade adenosquamous MBC present in which better outcome would be expected. This case series indicates that smaller tumor size at diagnosis or young age would carry a better prognosis. It also highlights the beneficial effects of early surgery in MBC.

In summary, early surgery on smaller tumor improves outcome, and this can be helped by recognition of unusual presentation of MBC. Most MBC is resistant to conventional chemotherapy. Contemporary chemotherapy with stem cell targeting is promising. Radiotherapy as one component of multimodality managements is a salvage for lumpectomy surgery in MBC.

## Figures and Tables

**Table 1:** Incidence, clinical and image presentation, biology, genetics, chemoradiation therapy, and prognosis of metaplastic breast carcinoma and invasive ductal carcinoma.

	Metaplastic breast carcinoma	Invasive ductal carcinoma
Clinicopathological features		
Incidence	0.2–1% [1]	85%
Age of presentation	Mean age 46–68, similar to TNBC [2, 14]	Mean age 45–60
Size (mean)	3.9–5.0 cm [1]	2.1–2.3 cm [1]
Axillary lymph node metastasis	Lower incidence, 6–28% [1, 2, 13]	34–50% [1, 2]
Hematogenous spread	More likely, preferentially affecting lung and brain (65%), and less likely in bone [1]	Less likely, preferentially affecting bone (60%), lung, and brain [1]
Stage at presentation		
Stage II or higher	>70% [3]	50% [3]
Stages III-IV	15.2–35.2% [1, 2, 14]	11–11.8% [1, 2]

Imaging	Benign (circumscribed, round, or oval on ultrasound, T2 hyperintensity on MRI) or malignant appearance [2, 13]	Malignant appearance (irregular or circumscribed with spicules)

Biomarkers		
ER/PR/HER2 triple negative	70–100% [13]	15% [4]
EGFR	Overexpression 93.9% [9], amplification 30% [2]	Overexpression 21.6% [9]
PIK3CA/PTEN mutation	47.4%/5.3% [8]	21.4%/2.3% [8]
Wnt/*β*-catenin deregulation	92% [7]	35% in IDC, 36% in benign breast [15]
p53 mutation and overexpression	50.9–63.8% [1, 2, 9]	28.8–38.8% [1, 2, 9]
Ki-67 (>=14%)	87.2% [1]	61.1–63.4% [1]

X-chromosome inactivation pattern	100% (4/4) clonal [5]	33% (4/12) mosaic (polyclonal) [6]

Chemotherapy		
Frequency [2, 13]	Twice likely with frequency of 53.4% in stage matched cases and 33–86% overall	Less likely with frequency of 42.1% in stage matched cases
Response to conventional taxane, anthracycline chemotherapy [2]	Neoadjuvant: 10% response Adjuvant: 10–17.6% response	Neoadjuvant: 11–45% response in TNBC Adjuvant: 21–75% response
Response to stem cell targeting adjuvant chemotherapy [10, 11]	40–42% complete and partial pathological response	
Response to cisplatin containing regimen	Adjuvant: 12% decreased relapse rate compared to taxane/anthracycline/cyclophosphamide regimen [1]	Neoadjuvant: 44.2% complete pathological response in TNBC compared to anthracycline (26.8%) and taxane (30.5%) group [4]

Radiotherapy [3]	Frequency of 38.6–49.1% 16.2% decreased risk of death in lumpectomy; 2.4% decreased risk of death in mastectomy	Frequency of 23% 17% decreased risk of death in lumpectomy

Prognosis		
Recurrent rate	60% usually within 5 years [13]	20% within a variable length of time [13]
Five-year survival	45.5%–63% [1, 13]	60.3% in TNBC and 71.2%–92% in IDC [1, 13]

IDC: invasive ductal carcinoma. TNBC: triple negative breast carcinoma.
